# Standardization of Misleading Immunoassay Based 25-Hydroxyvitamin D Levels with Liquid Chromatography Tandem-Mass Spectrometry in a Large Cohort Study

**DOI:** 10.1371/journal.pone.0048774

**Published:** 2012-11-01

**Authors:** Ben Schöttker, Eugène H. J. M. Jansen, Ulrike Haug, Lutz Schomburg, Josef Köhrle, Hermann Brenner

**Affiliations:** 1 Division of Clinical Epidemiology and Aging Research, German Cancer Research Center, Heidelberg, Germany; 2 Laboratory for Health Protection Research, National Institute for Public Health and the Environment, Bilthoven, The Netherlands; 3 Division of Preventive Oncology, German Cancer Research Center, Heidelberg, Germany; 4 Institut für Experimentelle Endokrinologie, Charité University Medicine, Berlin, Germany; University College Dublin, Ireland

## Abstract

**Background:**

The interest in vitamin D measurement has strongly increased in recent years. The best indicator for circulating vitamin D levels is 25-hydroxy-vitamin D (25(OH)D) which is often measured by different immunoassays. We demonstrate problems in comparability of measures by different immunoassays and the need for standardization in the context of a large population-based cohort study.

**Methods:**

25(OH)D was measured with the immunoassays Diasorin Liaison in 2006 in 5,386 women and in the context of another project with IDS-iSYS in 4,199 men in 2009–2010 (when the Diasorin Liaison was no longer available in the version utilized in 2006). Standardization was performed by re-measuring of 25(OH)D levels in 97 men and 97 women with liquid chromatography tandem-mass spectrometry (LC-MS/MS) to obtain linear regression conversion equations.

**Results:**

Applying a 30 nmol/L cut-off value for vitamin D deficiency would have resulted in 48.3% of women and 12.1% of men with vitamin D deficiency ahead of standardization. The large gender difference was strongly attenuated after standardization of the assays with only 15.7% of women and 14.3% of men with vitamin D deficiency. Standardization on average increased the 25(OH)D levels by 10.3 nmol/L in women and decreased 25(OH)D levels by 2.9 nmol/L in men.

**Conclusion:**

The standardization with LC-MS/MS revealed that much of the observed gender difference was only assay-driven and the extremely high proportion of 48.3% vitamin D deficient women proved to be an exaggeration of the old version of the Diasorin-Liaison immunoassay. Standardization of 25(OH)D immunoassay results by LC-MS/MS is recommended to improve their accuracy and comparability, provided the LC-MS/MS method itself is adequately validated and standardized.

## Introduction

The interest in vitamin D measurements is strongly increasing since low vitamin D status is no longer only known to be a risk factor for osteoporotic diseases [1] but has also been linked to the occurrence of a variety of other chronic diseases, such as cardiovascular diseases [2], diabetes mellitus [3] and several types of cancer [4]. It is widely acknowledged that serum 25-hydroxy-vitamin D (25(OH)D) is the best indicator for circulating vitamin D levels [5]. Laboratory procedures for 25(OH)D measurements include immunoassays, high performance liquid chromatography (HPLC) and liquid chromatography tandem-mass spectrometry (LC-MS/MS). Currently, automated immunoassays are the most popular method [6] for reasons of convenience, speed, turnaround and cost, especially for large sample sizes [7]. These are without any doubt advantages of automated immunoassays over LC-MS/MS and HPLC but the latter methods can have a higher sensitivity and selectivity, provided they are adequately validated and standardized [8], [9].

Results from different 25(OH)D immunoassays can vary strongly (differences of 10 nmol/L and more even in 25(OH)D levels below 50 nmol/L) because different standards and artificial calibrators are used by the suppliers [10]. This makes diagnostic and therapeutic decisions based on absolute cut-off values for vitamin D deficiency very difficult [11], [12] and strongly hinders comparability of results from epidemiological studies. We report serious problems in comparability of measures by different immunoassays and demonstrate the need for standardization in the context of a large population-based cohort study.

## Methods

### Ethics Statement

Informed consent was obtained by all study participants of the study and all clinical investigations have been conducted according to the principles expressed in the Declaration of Helsinki.

### Study design

Data shown originate from the ESTHER study (Epidemiologische Studie zu Chancen der Verhütung, Früherkennung und optimierten THerapie chronischer ERkrankungen in der älteren Bevölkerung [German]), an ongoing cohort study, details of which have been reported elsewhere [13], [14]. Briefly, 9,949 subjects, aged 50–74 years at baseline, were recruited by their general practitioners in the German federal state Saarland during a routine health check-up between 2000 and 2002. The ESTHER Study has been approved by the ethics committees of the Medical Faculty of the University of Heidelberg and the Medical Association of Saarland and is being conducted in accordance with the declaration of Helsinki. A signed statement of informed consent has been obtained from all participants included in the ESTHER study. Blood samples were taken during the health check-up, centrifuged, shipped to the study center and stored at −80°C.

### 25-OHD measurements

In 2006, in the framework of a project on women’s health, the automated Diasorin-Liaison analyzer (Diasorin, Inc., Stillwater, USA) was employed in the central laboratory of the University Clinic of Heidelberg to measure 25(OH)D levels from stored serum samples in 5,386 women. An intra-assay coefficient of variation (CV) from 8 to 21% and an inter-assay CV from 8 to 34% [6] have been ascribed to the assay. The lower detection limit in our laboratory was 15 nmol/L.

Funding was obtained for a new project in 2009 to measure 25(OH)D also from the stored baseline serum samples of male study participants. The Diasorin-Liaison method utilized for women was unavailable at this date because it had been replaced by the manufacturer by another method in 2007 [6]. It was decided to employ the automated IDS-iSYS (Immunodiagnostic Systems GmbH, Frankfurt Main, Germany). According to the information of the manufacturer, the assay has an intra-assay CV of <7.3%, an inter-assay CV of <8.9% and a lower detection limit of 9 nmol/L [6]. The 25(OH)D levels of 4,199 men were measured in 2010 in the laboratory of the Institut für Experimentelle Endokrinologie (Institute for Experimental Endocrinology), Charité University Medicine, Berlin.

### Comparison of the immunoassay results

To judge the comparability of the two immunoassays, the IDS-iSYS method was employed to measure 25(OH)D in 45 women for which 25(OH)D levels had already been determined with the Diasorin-Liaison method in 2006.

### Standardization with LC-MS/MS

From both women (in whom 25(OH)D was measured with Diasorin-Liaison) and men (in whom 25(OH)D was measured with IDS-ISYS) random samples of 100 study participants were drawn and re-measured with isotope-dilution LC-MS/MS (Waters ACQUITY TQ tandem quadrupole mass spectrometer (Waters, Milford, MA, USA)) in the Department of Clinical Chemistry, Canisius Wilhelma Hospital, Nijmegen, The Netherlands. The human serum calibrator of Chromsystem, Munich, Germany was utilized to standardize the LC-MS/MS. The LC-MS/MS assay takes part in the Vitamin D External Quality Assessment Scheme (DEQAS) and further details of standardization, precision and comparability to other assays have been described elsewhere [15].

**Figure 1:**
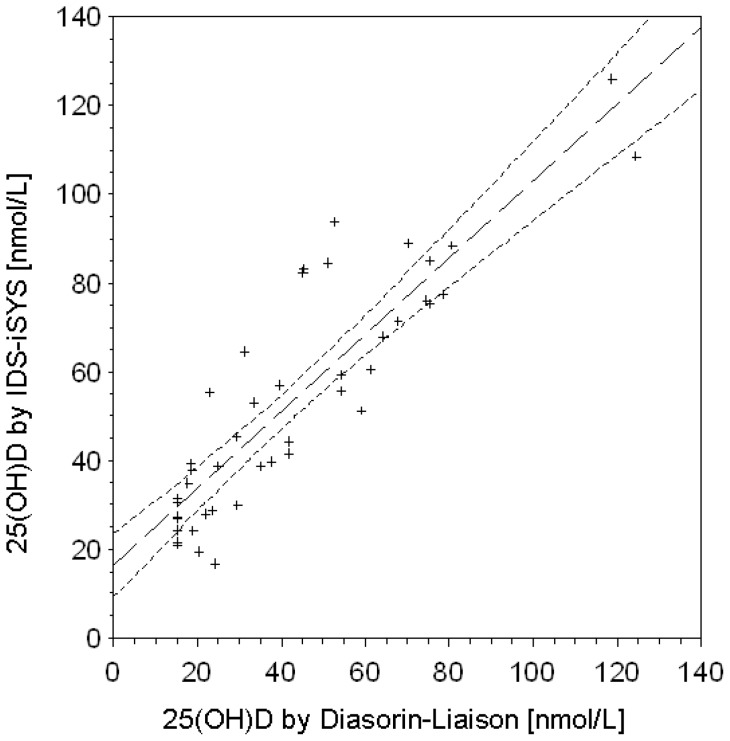
Correlation plot of Diasorin and IDS immunoassay 25(OH)D results in 45 subjects.

The correlations of the immunoassay results with LC-MS/MS were assessed and ordinary least squares linear regression equations were obtained and employed as standardization equations for 25(OH)D levels of the total cohort. All results and plots were generated with SAS, version 9.2 (Cary, NC, USA).

**Table 1:** Sex differences in baseline 25-hydroxyvitamin D (25(OH)D) levels before and after standardization with LC-MS/MS in the ESTHER study.

25(OH)D assay	n	Sex	Unstandardized		Standardized	
			Mean 25(OH)D levels [nmol/L]	Proportion with 25(OH)D <30 nmol/L (%)	Mean 25(OH)D levels [nmol/L]	Proportion with 25(OH)D <30 nmol/L (%)
Diasorin-Liaison	5,386	Female	36.2	48.3	46.5	15.7
IDS-iSYS	4,199	Male	60.2	12.1	57.1	14.3
Δ			24.0	36.2 percent points	10.6	1.5 percent points

## Results

### Comparison of the immunoassay results

In the random sample of 45 women with 25(OH)D measured by both the Diasorin-Liaison and IDS-iSYS immunoassay correlation was found to be high (r = 0.885, p<0.001, R^2^ = 0.783, Figure 1), but measurements by Diasorin-Liaison were on average 11.5 nmol/L lower than those by IDS-iSYS (p<0.001). Using non-standardized results from both methods would have suggested much higher mean 25(OH)D levels in men (60.2 nmol/L) than in women (36.2 nmol/L, p<0.001) in the total cohort (Table 1). Applying a cut-off value of 30 nmol/L (12 ng/ml) for vitamin D deficiency, that is currently recommended by the US-American Institute of Medicine [16], [17], would have resulted in proportions of 48.3% of vitamin D deficiency in women and 12.1% of vitamin D deficiency in men.

**Figure 2:**
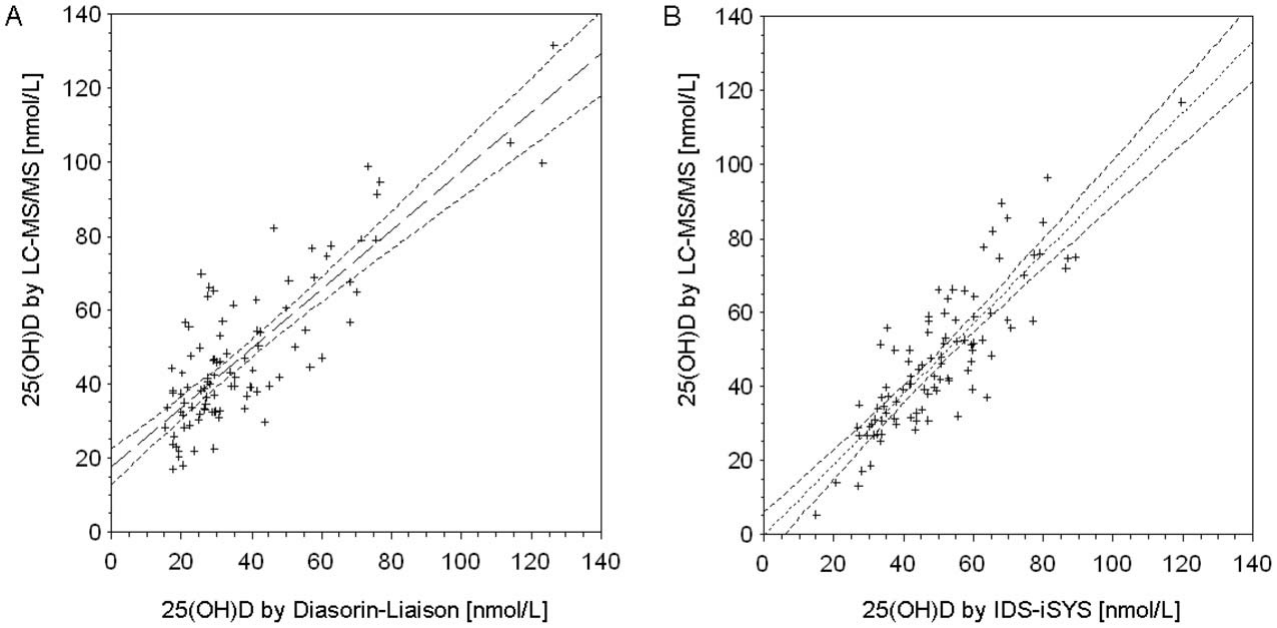
Correlation plot of Diasorin-Liaison (2A) and IDS-iSYS immunoassay (2B) 25(OH)D results with LC-MS/MS.

### Standardization with LC-MS/MS

To assess to what extent the gender difference was assay-related, both immunoassays were calibrated with 100 LC-MS/MS measurements for each assay. Three influential outliers (>2 standard deviations of the mean assay difference) were excluded for each assay which resulted in 97 pairs of 25(OH)D values for the standardization of each immunoassay.

The correlation plots of the Diasorin-Liaison and the IDS-iSYS with LC-MS/MS are shown in Figure 2. Both immunoassays are highly correlated with LC-MS/MS (Diasorin-Liaison: r = 0.832, p<0.001, R^2^ = 0.692; IDS-iSYS: r = 0.857; p<0.001, R^2^ = 0.734). However, measurements by Diasorin-Liasorin were on average 10 nmol/L lower than those by LC-MS/MS. By contrast, no difference in mean 25(OH)D levels was seen between measurements by IDS-iSYS and LC-MS/MS. The following standardization equations were obtained to convert 25(OH)D levels in the total cohort:

After conversion, the mean 25(OH)D levels increased from 36.2 nmol/L to 46.5 nmol/L for women (measured with Diasorin-Liaison) and decreased from 60.2 nmol/L to 57.1 nmol/L for men (measured with IDS-iSYS) (Table 1). The gender difference of 24 nmol/L was attenuated to 10.6 nmol/L and the proportion of vitamin D deficient women (measured with Diasorin-Liaison) decreased from 48.3% to 15.7% while the proportion in men (measured with the IDS-iSYS) increased from 12.1% to 14.3%.

### Corroboration of the standardization

In analogy to indirect comparisons of the efficacy of competing interventions in clinical trials the standardization of the Diasorin-Liaison measurements (in the following called method A), and the IDS-iSYS measurements (in the following called method B), by LC-MS/MS (in the following called method C) can be regarded as an indirect comparison of the performance of method A and B via their performance versus method C [18]. Indirect comparisons can be biased and their results should be corroborated by a direct comparison of A and B in the same participants [19]. We therefore performed a direct comparison of A and B in a random subsample of 45 women. The difference in means of method A and B was 11.5 nmol/L. This difference is completely assay-related because the measurements were performed in the same subjects. Assuming a successful standardization from method A and B by method C should level out this assay-related difference in means. This was tested by applying the obtained standardization equations to the 25(OH)D measurements of those 45 women for which measurements with both method A and B were available. Indeed, the mean 25(OH)D level determined by method A (Diasorin-Liaison) increased by standardization from 44.1 nmol/L to 52.8 nmol/L and the mean 25(OH)D level measured by method B (IDS-iSYS) decreased from 55.6 nmol/L to 52.6 nmol/L. The difference in means after standardization (0.2 nmol/L) was not statistically significantly different from zero any more (p = 0.921) which corroborates the standardization equations.

## Discussion

From a large population-based cohort study we showed exemplary data that 25(OH)D immunoassay results need to be standardized. The standardization with LC-MS/MS revealed that much of the observed gender difference was due to assay differences. The extremely high proportion of 48.3% of women with vitamin D deficiency was found to be an overestimate resulting from the use of an old version of the Diasorin-Liaison immunoassay. After standardization, the proportion of women with vitamin D deficiency was reduced to 15.7%. In contrast, the IDS-iSYS immunoassay results showed a quite good comparability with LC-MS/MS results.

The example from the ESTHER study is also a warning against comparisons of mean 25(OH)D levels from different studies if they applied different immunoassays. It has also been shown by others that differences in proportions of vitamin D insufficiency could strongly be influenced by assay differences [11], [12]. As demonstrated, the standardization to a standard method can exclude those assay differences. Therefore, the aim of vitamin D projects of the Consortium on Health and Ageing: Network of Cohorts in Europe and the United States (CHANCES) [20], the ESTHER study participates in, is to repeat the standardization as described for the ESTHER study in other cohorts to enable valid comparisons of the vitamin D status in different countries.

For these comparisons it is important that employed 25(OH)D assays measure both 25-hydroxyvitamin D_2_ (25(OH)D_2_) and 25-hydroxyvitamin D_3_ (25(OH)D_3_) because vitamin D_2_ dominates in supplementary vitamin products in the USA and vitamin D_3_ in Europe [21]. All assays employed in the ESTHER study measured 25(OH)D as a total of 25(OH)D_2_ and 25(OH)D_3_ [6], [15], [22]. The specificity for 25(OH)D_3_ was described to be close to 100% for all three employed assays. However, for 25(OH)D_2_ specificity was close to 100% for LC-MS/MS only, whereas specificities of the Diasorin-Liaison and IDS-iSYS immunoassays have been found to vary between 50 and 80% [6]. However, it should be noted that these specificity data may not apply for current releases of assay versions of the manufactures because of a lot of actions are going on in the field of 25(OH)D analytics to improve the 25(OH)D_2_ specificity. A further issue that can lead to 25(OH)D assays differences is the cross-reactivity of 25(OH)D assays with less biologically active metabolites, such as the 3-epimers of 25(OH)D_2_ and 25(OH)D_3_, that can result in an overestimation of total 25(OH)D concentrations [23]. All the methods used in this study, including LC-MS/MS, detect the 3-epimers to a variable degree. Although most manufacturers provide information on cross-reactivity for their latest 25(OH)D assay versions, this information is mostly unknown for older versions [8]. In general, the 3-epimers are currently a more important issue for LC-MS/MS than for immunoassays. A first LC-MS/MS method that can separate the epimers was described recently [24]. However, the LC-MS/MS method was applied to 5 individuals only and the epimer concentrations showed a large inter-individual variability [24]. However, both the variability in the 25(OH)D_2_ specificity and the 3-epimer cross-reactivity can likely explain assay differences only to a small extent like for example the small difference between the IDS-iSYS immunoassay and LC-MS/MS results. The observed large assay differences of the two aforementioned methods with the old version of the Diasorin-Liaison are more likely caused by insufficient standardization of the latter. Unfortunately, up to date there is no internationally acknowledged standard for 25(OH)D assays but research on such a standard is rapidly progressing [6], [8], [25].

In the United States, the National Health and Nutrition Examination Survey (NHANES) is probably the leading study in advancing standardization of 25(OH)D assays. Diasorin Radioimmunoassays employed in NHANES III and at later contacts showed a mean bias of 12% that was most probably caused by changes in reagents and calibration lots carried out by the manufacturer [26], [27]. Adjustment for these changes was done by re-measuring 150 NHANES III samples with the newer version of the assay and utilization of the obtained regression equation, following a similar approach of standardization as outlined for the 25(OH)D measurements in our study. Since November 2010, efforts are being made to re-calibrate 25(OH)D measurements from all NHANES contacts with LC-MS/MS within an international approach for standardization of 25(OH)D measurements in national surveys, called the Vitamin D Standarization Programme (VDSP), and the publication of results is planned for mid of 2013 [25], [28]. The VDSP developed the National Institute of Standards & Technology (NIST)-Ghent University reference measurement procedures (NIST-GHENT-RMPs) in the laboratories of these two institutions. The NIST-GHENT-RMPs measure total 25(OH)D, 25(OH)D_2_, 25(OH)D_3_ and the 25(OH)D_3_ 3-epimer with LC-MS/MS methods that have been standardized with the recently released NIST human serum based standard reference material (SRM) 972a and calibrated with the ethanol-based SRM 2972 [23], [25], [29].

The NIST-GHENT-RMPs have the potential to become the international standard procedure in total 25(OH)D measurement [8], [11], [30]. Nevertheless, for large studies and clinical routine, immunoassays will still have a future for reasons of convenience, speed, turnaround and cost [7], provided they are adequately calibrated. In recent years, suppliers of 25(OH)D immunoassays, like the companies Diasorin and IDS, updated their assays by calibration with LC-MS/MS and the latest publication that compared current versions of the Diasorin Liaison, the IDS iSYS and some other 25(OH)D assays with LC-MS/MS in a small number of randomly selected patient samples (n = 170) can raise optimism that the comparability of some immunoassays with LC-MS/MS may be acceptable already [8]. However, the authors also stated that there are still several 25(OH)D assays on the market that did not meet their minimum performance goals.

For large-scale studies like the ESTHER study and the NHANES survey with already performed 25(OH)D measurements in 10,000 and more study participants with older assay versions, the outlined re-measuring of a subsample with LC-MS/MS (e.g., n = 100) could serve as a cost-efficient and biological material saving model for standardization. The same principle will also be utilized by the VDSP to standardize past surveys to the NIST-GHENT-RMPs (“option 2”) [25]. The only difference to our approach is that we chose the LC-MS/MS method of another laboratory as the reference method. However, the international traceability of 25(OH)D measurements to the same LC-MS/MS method is important for the comparability of results and therefore the efforts of the VDSP to promote the NIST-GHENT-RMPs as the international standard, all 25(OH)D assays should be traceable to in future, are highly appreciated. To conclude, standardization with LC-MS/MS appears to be indispensible to ensure accuracy and comparability of 25(OH)D immunoassay results across studies.
